# A case of oropharyngeal *Ureaplasma urealyticum* infection in a human immunodeficiency virus positive bisexual male co-infected with human papilloma virus and *Treponema pallidum*

**DOI:** 10.1099/jmmcr.0.005132

**Published:** 2018-01-10

**Authors:** Nazneen Arif Suri, Sujeesh Sebastian, Deepika Yadav, Neena Khanna, Benu Dhawan

**Affiliations:** ^1^​ Department of Microbiology, All India Institute of Medical Sciences, New Delhi, India; ^2^​ Department of Dermatology and Venereology, All India Institute of Medical Sciences, New Delhi, India

**Keywords:** extragenital infections, men who have sex with men, non-gonococcal urethritis, oropharyngeal mycoplasmas, ureaplasma, co-infections

## Abstract

**Introduction:**

Management strategies for sexually transmitted infections (STIs) in their extragenital forms address *Neisseria gonorrhoeae* and *Chlamydia trachomatis* alone; whereas increased rates of isolation of other STI agents have been reported from various parts of the world. Their extragenital presence as a reservoir of infection emphasizes the need to screen and treat them at these sites.

**Case presentation:**

A 35-year-old human immunodeficiency virus 1 infected bisexual male presented with urethral discharge and multiple ano-genital warts. He was reactive for the venereal disease research laboratory (VDRL) test. He tested positive for *Ureaplasma* spp. both by culture and PCR at urethral and oropharyngeal sites, but was negative at the rectal site. The patient was successfully treated with doxycycline and penicillin, and was followed up with a test of cure at 6 weeks.

**Conclusion:**

In view of the disseminating infections that can be caused by *Ureaplasma* spp., it makes it important to screen for these infections even at non-genital sites, especially in the immunocompromised. STIs may be asymptomatic and can serve as a reservoir of infection in a population. This report should promote all efforts to formulate guidelines for extragenital screening of all STI pathogens.

## Introduction

Screening of extragenital sites, including the oropharynx and rectum, is an emerging practice based on recent studies highlighting the prevalence of infection at these sites [1]. The presence of sexually transmitted infections (STIs) at non-genital sites facilitates transmission and acquisition of STIs, including human immunodeficiency virus (HIV) [2]. Given that STIs are often asymptomatic, regular testing and timely treatment remains the cornerstone for optimal prevention of spread of these infections in the population. Hence, the Centers for Disease Control and Prevention (USA) recommends that all men who have sex with men (MSM) should be screened annually (or every 3–6 months, if at ‘increased risk’) for genital and extragenital infections with STI agents (HIV; syphilis; urethral, rectal and pharyngeal gonorrhoeae; and urethral and rectal *Chlamydia*) [3].

Management strategies, including investigations to address extra-genital *Chlamydia trachomatis* and *Neisseria gonorrhoeae* in both sexes, exist. Recommendations for screening of STIs at pharyngeal sites are there only for gonorrhoea. However, the same are lacking for other STIs, including genital mycoplasmas [3]. Many studies have reported the prevalence of pharyngeal *C. trachomatis*, usually asymptomatic with unclear implications [4]. Genital mycoplasmas have only rarely been isolated from pharyngeal sites in people, even in the presence of genital infections [5].

In addition to the ‘classical’ sexually transmitted pathogens, recent studies have also demonstrated a strong association between abnormal urogenital findings and the detection of *Ureaplasma urealyticum.* It is an important cause of non-gonococcal urethritis, accounting for nearly 5–26 % of all cases [6]. Though two species of *Ureaplasma* cause human infection, *Ureaplasma parvum* (biovar 1) and *U. urealyticum* (biovar 2), *U. urealyticum* is more significantly associated with symptomatic urogenital infections [7]. Here, we report a case of a HIV-positive bisexual male with oropharyngeal and urethral *U. urealyticum* infection, co-infected with human papilloma virus (HPV) and *Treponema pallidum.*

## Case report

A 35-year-old male presented to the STI clinic at the All India Institute of Medical Sciences, New Delhi, India, for evaluation of urogenital discharge and generalized lymphadenopathy involving the cervical, axillary and inguinal groups of lymph nodes. He had undergone surgical excision of ano-genital warts (AGWs) previously. He was bisexual, with multiple homosexual and heterosexual casual partners, with a history of unprotected receptive and insertive oral and anal sex. There was no history of past blood transfusion or intravenous drug abuse. He reported never being tested for HIV nor any other STIs. His medical history did not reveal any significant systemic abnormalities. On examination, he had scanty mucoid urethral discharge and multiple AGWs, with no lesions nor inflammation in the oropharynx and rectum. A Gram-stained smear of the urethral discharge under an oil-immersion objective of a microscope revealed 4–5 white blood cells, but no Gram-negative intracellular diplococci were seen. Microscopic examination of the urethral discharge was negative for any motile trophozoites, yeast cells and pseudohyphae. The patient was diagnosed clinically as a case of recurrent HPV infection with urethritis.

**Fig. 1.**
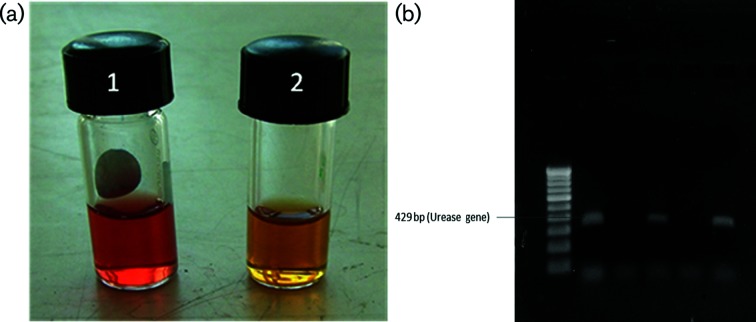
(a) Culture in PPLO broth. Tube 1 shows a culture positive result and tube 2 shows a culture negative result for *Ureaplasma* spp. (b) Gel electrophoresis picture of the PCR reaction targeting urease gene. Lane 1, 100 bp ladder; lane 2, positive control (NCTC 10177; urease positive); lane 3, negative control; lane 4, urethral sample (positive); lane 5, rectal sample (negative); lane 6, oropharyngeal sample (positive).

On investigation, his venereal disease research laboratory (VDRL) test was reactive (titre of 1 : 16) and his *T. pallidum* haemagglutination assay (TPHA) was positive. The patient was tested for HIV as per National AIDS Control Organisation (NACO) guidelines strategy 2B, in which three rapid tests were done for confirmation of diagnosis. CD4^+^ counts were estimated on a FACS Count microbead-based system, as recommended by the National Guidelines on the Enumeration of CD4 Lymphocytes [8]. The patient was HIV-1 positive, with a CD4^+^ lymphocyte count of 123 cells µl^−1^. He was seronegative for both hepatitis B surface antigen and anti-hepatitis C antibodies. Clinician collected urethral, rectal and oropharyngeal swabs were sent for detection of *N. gonorrhoeae, C. trachomatis* and genital mycoplasmas. Culture of the discharge for *N. gonorrhoeae* was negative on gonococcal agar with vancomycin, colistin, nystatin and trimethoprim sulfate, and so was the PCR assay for *C. trachomatis* targeting cryptic plasmid at genital, rectal and oropharyngeal sites. PCRs for *Mycoplasma hominis* and *Mycoplasma genitalium* were negative. Culture for *Ureaplasma* spp. was carried out in pleuro-pneumonia-like organism (PPLO) broth containing urea (Fig. 1a) and PCR was based on amplification of the urease gene (Fig. 1b) [9]. The isolate of *Ureaplasma* was further biotyped in a second PCR targeting the multiple banded antigen (MBA) gene. Species identification is based on an amplicon size of 408 bp for *U. parvum* (biovar 1) and an amplicon size of 448 bp for *U. urealyticum* (biovar 2) [10]. *Ureaplasma* was positive by culture and PCR in samples taken from the urethra and oropharynx, but negative in the rectal sample.

Histopathology of the warty lesion confirmed the diagnosis of AGWs. The patient was diagnosed to have oropharyngeal and urethral *U. urealyticum* infection, co-infected with HIV, *T. pallidum* and HPV. Contact tracing was attempted, but the patient refused to share any information about his partners.

The patient was treated with doxycycline, 100 mg twice daily for 7 days, and a weekly dose of 2.4 million IU benzathine penicillin for 3 weeks, for *Ureaplasma* infection and syphilis, respectively, as per Centers for Disease Control and Prevention guidelines for STI, 2015 [3]. Azithromycin and doxycycline have been recommended for non-gonococcal urethritis; however, considering the better response to doxycycline for extragenital *Chlamydia*, this was given. Penicillin remains the drug of choice for syphilis, with no resistance reported to date. The AGWs were treated with 10–20 % podophyllin resin, applied once a week for 8 weeks, and the patient was referred to an anti-retroviral therapy (ART) centre for management of the HIV infection. On evaluation at 6 weeks, he reported a cessation of the urethral discharge with healing of the warty lesions. A post-treatment, oropharyngeal swab and first-void urine sample of the patient were negative for *Ureaplasma* spp. both by culture and PCR. The patient was counselled for safe sex practices and advised to have regular follow-ups.

## Discussion

Extragenital *Ureaplasma* infections have been rarely reported and, to the best of our knowledge, this is the first report of oropharyngeal *Ureaplasma* infection from India. There has been one recent report of rectal *Ureaplasma* infection found in a culture from an MSM individual [11]. Globally, there are few studies of pharyngeal infection with *Ureaplasma* spp. Isolation from the pharynx of both men and women seeking treatment at several clinics in the USA yielded *Ureaplasma* prevalence rates of 14.8 %, including *U. parvum* (biovar1) and *U. urealyticum* (biovar 2), which were not distinguishable at that time [5]. In a study from Japan, the prevalence of *U. parvum* and *U. urealyticum* in the pharynx of Japanese female sex workers practicing fellatio on their clients was 0.2 and 0.7 %, respectively, whereas the prevalence in the genitals was 40.4 and 10.2 %, respectively. Though presence in the pharynx was not significantly associated with presence in the genitals, a history of performing fellatio was significantly associated with *U. urealyticum* pharyngeal infection [12]. Even though the significance of isolation of *U. urealyticum* from the oropharynx is not established, it may serve as an extragenital reservoir. It has been established that the risk of retroviral transmission is increased in the presence of co-infections with organisms that breach the epithelial barrier [13]. Since *Ureaplasma* infections are potentially mucosa disrupting, they may similarly increase the risk of HIV transmission, and detection may be important even if the patient is asymptomatic. However, the low CD4^+^ count in our patient, which suggests a long-term HIV infection, could be responsible for the various infections acquired. The generalized lymphadenopathy in the patient could possibly be a manifestation of HIV infection. There were no symptoms of any primary or secondary syphilis, so diagnosis of latent syphilis was made, and the patient was treated accordingly.

Barbee and colleagues have previously observed that nearly a third of all cases of symptomatic gonococcal and non-gonococcal, non-chlamydial urethritis (NGNCU) among MSM attending an STI clinic was probably acquired through oral sex [14]. A limitation of their study was their inability to identify the aetiological agents of NGNCU, as these agents were not tested for routinely in their clinic. In our patient, the same organism, *U. urealyticum* (biovar 2), was isolated from the urethra as well as the oropharynx, supporting acquisition through orogenital contact. However, this might be two independent transmissions as well. The proximity of the oropharynx to the lungs is of concern, as evident in recent reports, in which disseminated *Ureaplasma* resulting in hyperammonaemia caused fatal consequences in immunocompromised patients [15, 16].

Screening of all patients who present to the STI clinic for multiple STIs is now recommended and this case highlights the importance of screening all patients with high-risk behaviour for multiple co-pathogens infecting the genital tract. Early detection and treatment of sexually transmitted pathogens in HIV-infected patients would prove prudent to control retroviral transmission. However, commercially available nucleic acid amplification tests (NAATs) have not been validated for samples from extragenital sites. New standard use of commercial NAATs for *N. gonorrhoeae* and *C. trachomatis* alone may be missing out on infections that culture methods or expanded NAATs would otherwise identify. A multiplex NAAT does not include testing for *Mycoplasma* or *Ureaplasma* spp. and, thus, these infections will not be detected. Expansion of the STI test panel in commercial systems and additional specimen source sampling within a comprehensive programme will increase identification of STI carriers. Addressing currently hidden genital mycoplasmas by extragenital testing of more persons would reveal many more reservoirs of infections. There is little evidence on the specificity or sensitivity of NAATs for *U. urealyticum* at extragenital sites. However, our patient tested positive for *U. urealyticum* by both culture and PCR at both the sites, thereby ruling out the possibility of false-positive results. An arbitrary choice of 6 weeks was made as there are no published data or guidelines on the ideal timing for a *U. urealyticum* test of cure at an extragenital site.

This report is suggestive of the idea that failure to identify and eradicate *Ureaplasma* spp. from the oropharynx may play a critical role in its transmission in MSM. Considering the role of disseminated *Ureaplasma* in immunocompromised patients, they are of great concern. Furthermore, this report should add momentum to the efforts to promote widespread pharyngeal screening for other pathogens. It is also important to conduct investigations to define the yet unidentified organisms causing infections of the oropharynx that may be responsible for a significant proportion of cases of NGNCU.
